# Prenatal Loud Music and Noise: Differential Impact on Physiological Arousal, Hippocampal Synaptogenesis and Spatial Behavior in One Day-Old Chicks

**DOI:** 10.1371/journal.pone.0067347

**Published:** 2013-07-05

**Authors:** Tania Sanyal, Vivek Kumar, Tapas Chandra Nag, Suman Jain, Vishnu Sreenivas, Shashi Wadhwa

**Affiliations:** 1 Department of Anatomy, All India Institute of Medical Sciences, New Delhi, India; 2 Department of Physiology, All India Institute of Medical Sciences, New Delhi, India; 3 Department of Biostatistics, All India Institute of Medical Sciences, New Delhi, India; University of New England, Australia, Australia

## Abstract

Prenatal auditory stimulation in chicks with species-specific sound and music at 65 dB facilitates spatial orientation and learning and is associated with significant morphological and biochemical changes in the hippocampus and brainstem auditory nuclei. Increased noradrenaline level due to physiological arousal is suggested as a possible mediator for the observed beneficial effects following patterned and rhythmic sound exposure. However, studies regarding the effects of prenatal high decibel sound (110 dB; music and noise) exposure on the plasma noradrenaline level, synaptic protein expression in the hippocampus and spatial behavior of neonatal chicks remained unexplored. Here, we report that high decibel music stimulation moderately increases plasma noradrenaline level and positively modulates spatial orientation, learning and memory of one day-old chicks. In contrast, noise at the same sound pressure level results in excessive increase of plasma noradrenaline level and impairs the spatial behavior. Further, to assess the changes at the molecular level, we have quantified the expression of functional synapse markers: synaptophysin and PSD-95 in the hippocampus. Compared to the controls, both proteins show significantly increased expressions in the music stimulated group but decrease in expressions in the noise group. We propose that the differential increase of plasma noradrenaline level and altered expression of synaptic proteins in the hippocampus are responsible for the observed behavioral consequences following prenatal 110 dB music and noise stimulation.

## Introduction

Environmental stimuli influence the development of different sensory systems. The importance of light exposure during development of the visual system has been extensively studied, demonstrating that modifications of visual input or its deprivation result in differential outcomes in brain structure and function [1]–[6]. Here we report the findings on the effects of prenatal auditory stimulation at a higher sound pressure level on the development of hippocampus of neonatal chick at biochemical and behavioral level. Auditory stimulation by music or species-specific calls influences the cognitive performance of humans and different animals [7]–[12]. Early maturation of the auditory system [13]–[15] and the development of species typical perceptual preference is observed in birds following exposure to species own typical call [16], [17]. Our previous studies showed improved morphological and biochemical changes in chick hippocampus and facilitation of spatial orientation and learning at 12 hour post hatch consequent to prenatal sound stimulation by species-specific calls and sitar music at 65 dB [18]–[21]. The facilitation of learning was observed even at 24 hour post hatch with no further improvement in successive trials following the same sound enrichment protocol suggesting an early maturation of synaptic connectivity following prenatal sound stimulation. Significant memory retention at 72 and 120 hour post hatch with a transient impairment at 24 hour post hatch following prenatal music stimulation was observed [22]. The avian hippocampus is involved in spatial learning as well as in memory [23]–[25] and is connected to the auditory pathway directly as well as indirectly [26]–[29].

It is noteworthy that both sounds used for auditory enrichment in our earlier studies were rhythmic in nature and of moderate sound pressure level (65 dB) which had beneficial influences on the spatial and cognitive abilities of neonatal chicks. However, the prenatal music (rhythmic sound) or noise (arrhythmic sound), when given at a higher sound pressure level (110 dB), may affect the cognitive abilities in a different manner, for which little information is available.

In today’s world pregnant women are often exposed to high decibel music as well as noise. This exposure may influence the developing fetus and subsequently alter the cognitive abilities of the newborn babies. The paradigm to study the effects of prenatal high decibel sound stimulation on neuroanatomical or biochemical changes is beset with limitations in human subjects. In view of this, the domestic chick (*Gallus gallus domesticus*) was chosen as the experimental model in the present study, as it is precocious [3], has a fairly well developed auditory system at birth like humans and can respond to air-borne sound during the embryonic period [30]. In the basilar cochlear papillae of the chick, the afferent synapses appear on the hair cells by the embryonic day E8-E11 [31] and the auditory Field L starts differentiating from E8 onward [32]. The acoustically evoked potentials to intense auditory stimuli can be recorded in the brainstem by E11-E12 [33]. Therefore, in the present study the auditory stimulation was provided from E10 onward immediately before the development of physiological functions of the peripheral auditory areas and the brainstem auditory nuclei.

It has been suggested that the advanced ability of music discrimination is present even in species that are evolutionarily distant from humans, including birds [34]. Be it music or species-specific call, it is the rhythmicity, pattern, frequency and ethological relevance of the sound to the animal that alters learning and memory [35]–[37]. Postnatal short-term exposure to rhythmic and patterned sound stimuli at 66 dB potentiates long-term memory consolidation in chicks whereas isochronous or unpatterned sound at the same sound pressure level does not show any effect [36], which confirms that the characteristic features of sound are important in mediating the effect. The facilitation of cognitive abilities by rhythmic auditory stimuli is considered to be mediated via an increased physiological arousal [38], [39], which is an indispensable requirement for long term memory consolidation [40]. Involvement of noradrenaline in memory formation via physiological arousal is well studied and surprisingly it is found that while moderate level of exogenously administered noradrenaline improves cognitive functions, in excess it impairs the performance [41]. Prenatal music (rhythmic sound) and noise (arrhythmic sound) stimulation may influence physiological arousal and thus plasma noradrenaline level in post hatch day one (PH1) chicks. The blood brain barrier is not fully developed until one month post hatch in chicks [42] and thus plasma noradrenaline can reach and affect different brain regions during that period which subsequently may influence the learning and memory of PH1 chicks. Our current study, thus, sought to address this hypothesis by measuring the plasma noradrenaline level and assessing spatial learning and memory in PH1 chicks following prenatal high decibel, music and noise stimulation.

Corticosterone level has been widely used as a marker of stress. A positive correlation of stress and plasma corticosterone level is established in chicks [43]. Exposure to high decibel sound may produce stress to embryos and to assess that, plasma corticosterone level of PH1 chicks was measured.

To understand the cellular mechanisms of spatial orientation, learning and memory, it becomes relevant to study different biochemical changes in chick hippocampus following prenatal loud sound exposure. Synaptophysin is an integral membrane protein and a major component of pre synaptic vesicles in neurons as well as of neuroendocrine cells [44]–[46]. Studies have shown that enriched environment can significantly increase synaptophysin expression in the hippocampus, which is associated with improved spatial memory in aged mice [47]. On the other hand, a decreased expression of synaptophysin is reported in rat hippocampus after restraint stress [48]. Synaptophysin therefore, plays an important role in modulating synaptic plasticity. Postsynaptic density protein-95 (PSD-95) is one of the most abundant proteins found in the post-synaptic membranes of glutamatergic excitatory synapses [49]. It regulates hippocampal and cortical long-term potentiation, plays a critical role in controlling the synaptic strength and activity-dependent synaptic plasticity and therefore has an indispensable role in learning [50], [51]. We therefore, hypothesize that alterations in the expression of the pre- and post-synaptic proteins in the hippocampus of PH1 chicks following prenatal high decibel auditory stimulation would explain the changes in behavioral outcome.

In this paper, we report the effect of prenatal high decibel (110dB) music and noise stimulation on the levels of plasma corticosterone and noradrenaline, synaptic proteins- synaptophysin and PSD 95 in the hippocampus, as well as spatial orientation, learning and memory of PH1 chicks.

## Materials and Methods

### Subject

White Leghorn domestic chicks (*Gallus gallus domesticus*) were used as the experimental model. Fertilized zero day eggs of healthy chicks, weighing 50–60 g, were obtained from a local registered poultry farm, and incubated in a double insulated egg incubator (Widson Scientific Works Ltd., New Delhi).

### Incubation Conditions

The eggs were incubated under controlled conditions as described earlier [52]. Briefly, the temperature of the incubator was maintained at 37°C (±1°C) and humidity at 70% (68–72%). The incubator had a forced draft of air for aeration and uniform level of temperature and humidity throughout the chamber. Photoperiod of 12∶12 hour day: night cycle and tilting of eggs four times a day were controlled electronically with automated timer devices attached to the incubator throughout the incubation period (21 days). Tilting of eggs was done to mimic the natural conditions, where the mother hen turns the eggs at regular interval throughout the incubation period [3]. To ensure that the embryos receive sound, a window of approximately 3 mm diameter was made by removing a part of egg shell over the air sac at the animal pole on day 9.5 of incubation, keeping the inner shell membrane intact. A background sound of 40 dB originating from the compressor of the incubator was audible two or three times per hour. This sound was not possible to eliminate and could sometimes coincide with the playback of music or noise. However, this co-occurrence of background sound (40 dB) and music/noise (110 dB) did not make any alteration in the total sound pressure level output as calculated using the following formula:
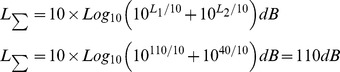



Where L_Σ_ = Total sound pressure level, L1 = sound pressure level of music/noise in our study and L2 = sound pressure level of the background noise emanating from the compressor of the incubator.

### Auditory Stimulation Protocol

The embryos were given auditory stimuli at 110 dB through two speakers affixed to the opposite walls of the incubating chamber (82 cms apart) and connected to a JVC double-deck sound system with auto-reverse facility. In the current industrial and recreational milieu, exposure to loud sounds during the day as well as night has become a significant problem [53]–[55]. The loudness of sound at rock concert or farm machinery is 110–120 dB [56]. The sound pressure level in a discotheque is usually 110 dB according to National Institute for Occupational Safety and Health (NIOSH), USA. Also the vehicle horn sound used in this study was recorded during traffic hours which showed a sound pressure level of 110±3 dB. It is also known that the occupational time schedule of exposure to loud sounds is generally 6–8 hours per day in our daily life. Hence, to simulate these conditions, the auditory stimulation in the present study was provided at 110 dB for 15 minutes per hour, over a period of 24 hours (total 6 hours per day) from the embryonic day 10 (E10) until hatching. In chicks, the auditory system develops during the second half of the incubation period and is adequately mature at birth. Therefore in the present study, the sound was given until hatching so that the chronic effects of sound exposure over the entire developmental period could be studied.

The protocol and number of animals used in the experiment was approved by the Institution Animal Ethics Committee of All India Institute of Medical Sciences, New Delhi, India (permit number: 445/IAEC/08).

**Figure 1:**
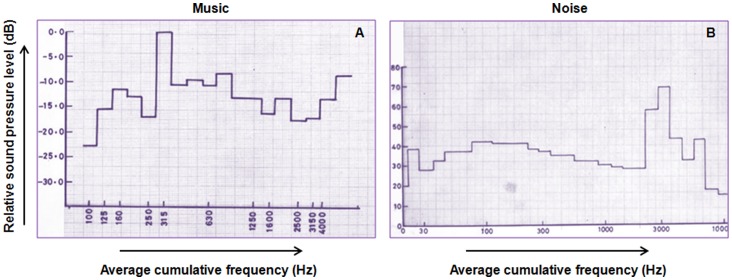
Graphs showing average cumulative 1/3 octave band frequency spectrum (Hertz) with relative sound pressure level (dB) of sitar music (A) and noise (B). In the X axis the contributions of different 1/3 octave frequency bands are seen and the Y axis of the graph represents the unique sound pressure level for each 1/3 octave frequency band which yield the output sound pressure level of 110 dB. Note that the variations of sound pressure levels of the different frequency bands are more in the music stimulus (**A**) as compared to the noise stimulus (**B**).

**Figure 2:**
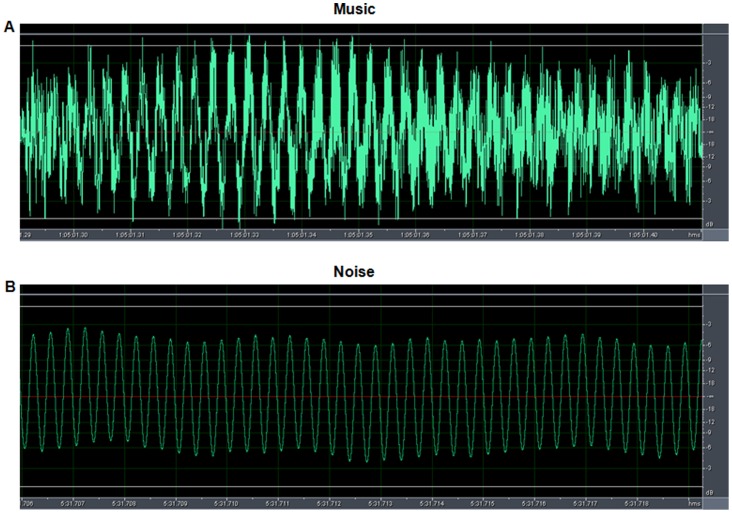
Time expanded wave form of music (A) and noise (B) stimulus. (**A**) Note the complex wave form of the music stimulus with variations in wavelength with time. (**B**) The simple wave form of noise stimulus shows no variations in wave length with time.

**Figure 3:**
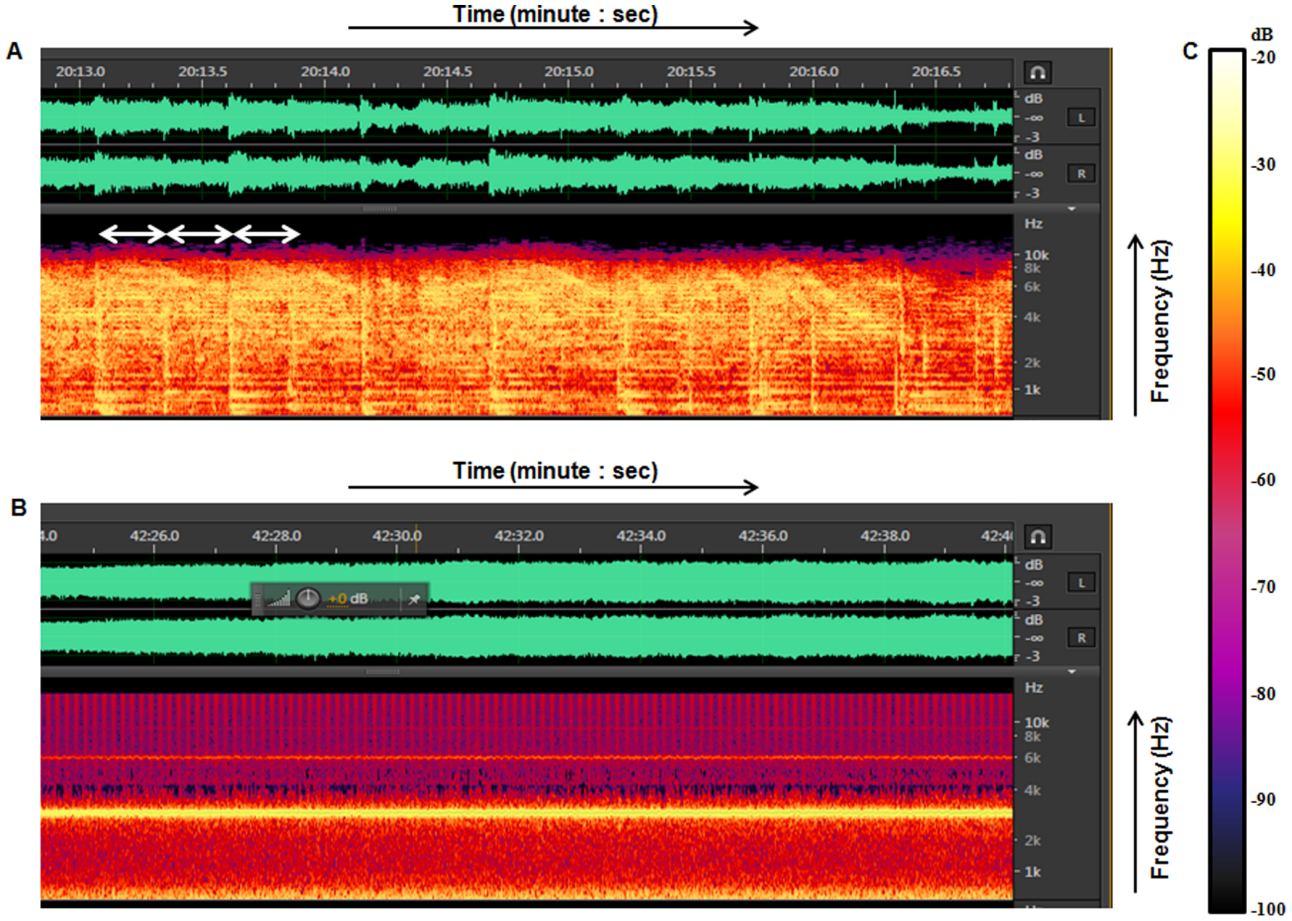
Spectrograms showing sound energy content of music (A) and noise (B) stimulus expressed as function of frequency and time. (**A**) Note that in the music stimulus, the high sound energy (bright yellow colour) is distributed over the entire frequency range and a particular pattern is repeated over time (note the double arrows ↔). (**B**) In the noise stimulus the high sound energy (bright yellow colour) is centered around 2.7 kHz. (**C**) Colour panel encodes the range of sound energy: colour toward white denotes high energy and toward black denotes less energy.

The sound recordings of the noise (vehicle horn sound) and music (sitar) were used for auditory stimulation. The piece of sitar music used in the present study was based on raga-“Alhaiya Bilawal” which is a fast tempo melodic composition, usually set to allegretto speed or faster (235 BPM). It is complex in its phraseology and has all the notes of the Western major scale [57] with additionally a flat *Ni* in the descent and *Ma* being omitted from the ascent [58]. The typical phrases and scales used for *raga* elaboration in a Alhaiya-Bilawal performance are- mnDP, RGPmG, NDNS, DnDP, GRGP, DG and S R G m P D n N S respectively [58]. The physical properties of the sound recordings were analyzed at the National Physical Laboratory (Council of Scientific and Industrial Research, India) and the Indian Institute of Technology (New Delhi, India). With the help of an AD-3521 Fast Fourier Transformation Analyzer, the frequency of sound at every time point of the wave pattern of auditory recordings was estimated. The cumulative frequency range of these stimuli in 1/3 octave bands and relative amplitude modulation were evaluated using a Real Time Bruel and Kjaer Analyzer. The frequency of the music stimulus ranged between 100 to 4000 Hz and that of the noise stimulus was within 30 to 3000 Hz with a peak at 2.7 KHz (Fig. 1A–B). The time expanded wave forms of the sounds were recorded by the Adobe audition software (version CS6, India). The noise recording showed simple waves with a constant wave length whereas the sitar (a string instrument) music demonstrated complex wave form (Fig. 2A–B). Spectrogram of the music recording showed the distribution of the high energy sound across the entire frequency range in comparison to the noise spectrogram where the highest energy was centred at the peak frequency, i.e., around 2.7 KHz (Fig. 3A–C). The variations in sound energy with frequency and time scale represented a rhythmic pattern in music stimulus whereas in noise the energy did not vary over time for a particular frequency giving it a relatively continuous and arrhythmic pattern. The only physical property of these two acoustic stimuli which was matched and controlled was the output of 110±3 dB of sound pressure level inside the incubator, confirmed with a sound level meter (Bruel and Kjaer). In order to assess the effects of music stimulation on the animals it is a prerequisite that the frequency range of the musical stimulus should be within the range that the species naturally communicates and hears [34]. In the present study, the frequency range of the music piece was within the range of the chick species specific calls (100–6300 Hz).

### Experimental Groups

The eggs were divided into following three groups –

Group I: Control- The eggs were incubated under usual incubation conditions. No additional sound stimulation was given and this group was considered as the control.

Group II: Rhythmic sitar music- Embryos were given auditory stimulation by the sitar music within a frequency range of 100–4000 Hz at 110 dB sound pressure level (SPL) from E10 until hatching. This group was termed as “music” group.

Group III: Arrhythmic noise- Here the embryos were exposed to arrhythmic loud sound of frequency range 30–3000 Hz at 110 dB SPL from E10 till hatching. This group was termed as “noise” group.

### Collection of Blood Plasma and Estimation of Corticosterone and Noradrenaline Levels

Post hatch day 1 (PH1) chicks (N = 30 Males+30 Females/group) were anesthetized with anesthetic ether and placed on a flat surface, positioning the ventral side upright. The keel (breast bone) was dissected to expose the heart and a two milliliter syringe was inserted at an angle of 45° to the body. About 1–2 ml blood was withdrawn from the heart of each chick and collected in heparinized vials. Sex of each chick was examined after collection of blood. The blood samples were centrifuged to separate the plasma and stored at −80°C until use. To find out whether prenatal chronic auditory stimulation at high sound pressure level (110dB) produced stress to the embryos, the plasma corticosterone level was measured by ELISA (DRG kit, Springfield, New Jersey, USA). All samples and standards were run in triplicates. The optical density was measured at 450 nm in a microtiter plate reader (Bio-Rad, Hercules, CA, USA) and the plasma concentrations of corticosterone were measured by MPM 6 software (Bio-Rad, Hercules, CA, USA).

To comprehend auditory stimulation induced physiological arousal, if any, which can influence the learning and memory of PH1 chicks (N = 15 Males+15 Females/group), plasma noradrenaline level was also measured by commercially available ELISA kit from LDN (Am Eichenhain, Nordhorn, Germany).

One way parametric ANOVA followed by post hoc Bonferroni test was performed to statistically compare the data among the males of all 3 groups and females of the 3 groups separately. Within a group, comparisons between males and females were made using Student’s t-test.

### Tissue Processing

The PH1 chicks from control (Group I) and auditory stimulated groups (Group II and III) were anesthetized with anesthetic ether and sacrificed by decapitation. Brains were dissected out, the forebrains cut and fixed in 4% paraformaldehyde for 7 days at 4°C for immunohistochemistry. For Western blotting, the forebrains were snap-frozen in liquid nitrogen and stored at −80°C until use.

### Immunohistochemistry Procedure

Immunohistochemistry was performed to localize the synaptic proteins: synaptophysin and PSD 95 in the hippocampus of PH1 chicks. Paraformaldehyde fixed forebrains (N = 18, 6 brains/group) were washed in 0.1 M phosphate buffer and then cryoprotected in 15% and 30% sucrose solution consecutively. Coronal sections of 14 µm thickness were cut using a Microm-HM 525 Cryostat (Microm International GmbH, Walldorf, Germany) and mounted on gelatinized slides. Sections were quenched with 0.3% H_2_O_2_ in 80% methanol for blocking endogenous peroxidase activity followed by incubation in 10% normal horse serum to block non specific binding. Sections were then incubated in primary antibodies: anti-synaptophysin and anti-PSD 95 (dilution: 1∶500, mouse monoclonal, Chemicon, Millipore, CA, USA) for 48 hrs at 4°C. The cross reactivity of the antibodies to chick brain epitopes has been shown earlier in another study [18]. After three washes, the sections were treated with biotinylated secondary antibody (dilution: 1∶200, anti-mouse, Vector laboratories, Burlingame, CA, USA) for 24 hr at 4°C and then in avidin-biotin-peroxidase complex (Vectastain Elite Kit, Vector Laboratories, Burlingame, CA, USA) for 2 hr. The binding sites of antigen-antibody interaction were visualized by 0.06% 3, 3′-diaminobenzidine tetrahydrochloride as a chromogen (Sigma Chemicals Co., MO, USA) and 0.06% H_2_O_2_ as the substrate. Sections from the control and the two experimental groups were processed simultaneously to maintain identical working conditions. For negative control, sections from the hippocampus were processed by the same protocol, excluding the primary antibody incubation step; the sections from chick cerebellum were used as positive control to confirm the specificity of the antibodies.

### Western Blotting and Quantitation

Western blotting for synaptophysin and PSD 95 was performed to see the quantitative changes in the expression of both synaptic proteins among the 3 groups (N = 18, 6 brains/group). The forebrain specimens that were snap-frozen and stored at −80°C were cryosectioned at 10 µm thickness using a cryostat (Microm-HM 525, Thermo Scientific, Germany). The tissues were trimmed while sectioning to include only the dorsomedial and ventral parts of the hippocampus [59] and were solubilised in 200 µl of protein extraction buffer [3 M Tris-HCL pH 8.0, 5 M NaCl, Nonidet P-40 (0.01%), Glycerol (0.1%), MilliQ-water, 100 mM Na_3_VO_4_, 0.05% Protease inhibitor cocktail (Sigma Chemicals Co., St Louis, MO, USA)]. The buffer with crushed sections of hippocampus was kept at 4°C for 1 hour and then centrifuged at 10,000 rpm for 30 min at 4°C [60]. The supernatant was collected and stored at −80°C until use. Before proceeding for Western blotting, the total protein concentrations of the samples were determined by Bradford’s method (1976). Equal amount of protein (30 µg) was loaded per lane on 12% SDS polyacrylamide gels and electrophoresed using mini protean tetracell gel apparatus (Bio-Rad, Hercules, CA, USA). The proteins were then transferred onto polyvinylidine difluoride (PVDF) membranes (Bio-Rad, USA). The membranes with transferred proteins were first treated with 5% BSA for 2 hr at room temperature to block non specific binding and then incubated in primary antibodies: anti-synaptophysin, anti-PSD 95 (1∶1000, mouse monoclonal, Chemicon, Millipore, USA) and anti-α-tubulin (1∶5000, mouse monoclonal, Sigma Chemicals Co., St Louis, MO, USA) for 12 hr at 4°C. The blots were then incubated in horse anti-mouse secondary antibody (1∶2000, Vector Laboratories, CA, USA) followed by avidin-biotin-peroxidase complex for 2 hr at room temperature in each step. The blots were then visualized by 0.06% DAB tetrahydrochloride using 0.06% H_2_O_2_ as the substrate. All incubations were performed in a temperature controlled shaker (Widson Scientific Works Ltd., New Delhi, India). Protein extracted from PH1 chick cerebellum was used as positive control and α-tubulin was used as a loading control for proteins in each lane. The selectivity of the antibodies was confirmed by performing Western blot of proteins extracted from chick and rat hippocampal tissue.

Densitometric analysis of Western blots on the coded specimens was performed using Quantity 1 software of the gel documentation system (Bio-Rad, USA). All blots were scanned simultaneously and normalized with α-tubulin. One way parametric ANOVA followed by post hoc Bonferroni test was performed to statistically compare the data among the control and the auditory-stimulated groups.

### Tests for Behavior

i **T-maze test.** T- maze test was performed to evaluate the spatial orientation, learning and memory behavior of PH1 chicks. These innate abilities are critical for their survival and can be shaped by the environmental conditions during incubation [3]. The apparatus consists of a T-arm with an isolation chamber to visually isolate a chick from its brood mates. The isolation chamber (21 cm × 21 cm) leads to a T corridor (21 cm × 7c m) with 7 cm × 7 cm short perpendicular arms. A 10 cm × 10 cm mirror was placed at the junction of the T corridor to facilitate movement of the chick from the isolation chamber towards the brooder area. One of the short perpendicular arms of the apparatus faced the brooder area. The brooder area was illuminated with bright lamp suspended immediately above it. The apparatus was placed in a small room at an ambient temperature and illumination.

The procedure as standardized by Gilbert et al. [61] was used to train the chicks on T-maze and conducted in a sound attenuated room at room temperature (27–28°C). Before training, all chicks were placed in the brooder area for an hour. Then one chick at a time was selected for training and placed in the center of the isolation chamber facing away from the T corridor. The time taken by the chick to exit the isolation box i.e. isolation box latency (IBL), the exploration time (ET) in the T-arm and the total time (TT) to reach the target (brooder area) was recorded using a stop watch. A cut off criterion of 600 sec was set. Three such trials were conducted with an interval of 10 min between each trial for each chick. First trial was done at 12 hour post hatch to test their spatial orientation in a novel environment, followed by two successive trials to test their spatial learning. The ability of the chicks to memorize the task was tested by repeating the T-maze test 24 hr after the last trial. The experimenter was blind to the stimulation protocol. A total of 90 chicks (control N = 30 and experimental N = 30+30) were tested to achieve statistically significant results.

Parametric tests of one way ANOVA and repeated measure ANOVA were performed for inter- and intra-group comparison, respectively. Post hoc analysis was performed with Bonferroni test. The memorizing ability was assessed using a paired t-test.

ii **Open field test.** To further analyze the exploratory and locomotor behavior, the open field test was performed on post hatch day 1 chicks. The open field was a square observation chamber (50×50×35 cm) whose floor was covered with black paper for effective contrast. The arena was subdivided into an inner (25×25 cm) and outer zones using the software. The chicks were tested one at a time. After placing the chick in the centre of arena, its behavior was observed and recorded through a camera positioned above the open field (Noldus Ethovision XT- version 8.0). The session lasted for 10 min after which the chick was returned to its brooder area. The following parameters were recorded: total distance moved, duration of stay in the inner or outer zone, latency of entry into outer zone, frequency of entry into inner or outer zone and total time spent moving or at rest.

The results were evaluated by one way analysis of variance (ANOVA) followed by Bonferroni post hoc test.

## Results

### Plasma Corticosterone Level

**Figure 4:**
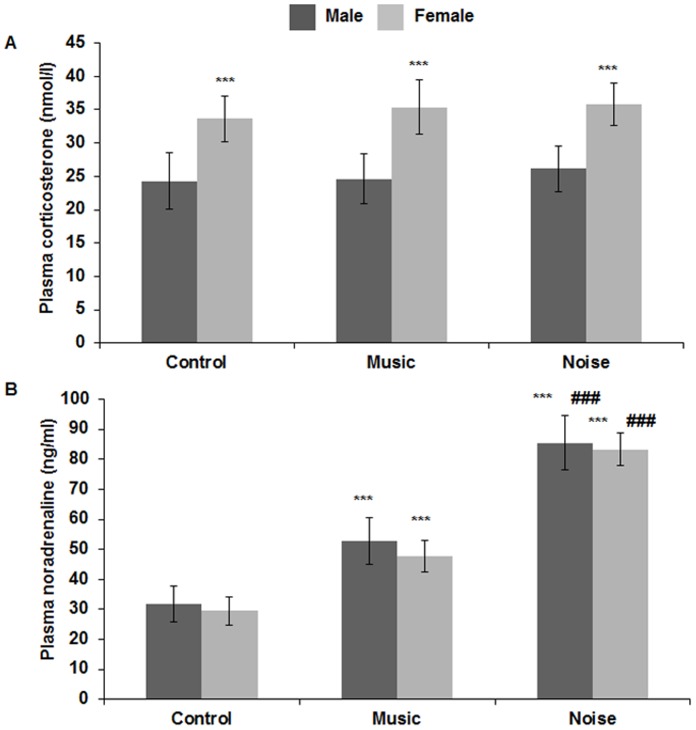
Changes in plasma corticosterone and noradrenaline level. (**A**) Histogram shows plasma corticosterone level (nmol/l) at PH1 in male and female of control and experimental groups. No significant alteration of plasma corticosterone level is observed in either of the sexes following music or noise stimulation, although females show significantly higher level as compared to males in all three groups. * indicates comparison of corticosterone level between male and female. (**B**) Histogram shows plasma noradrenaline level (ng/ml) at PH1 in male and female of control and experimental groups. Moderate increase of plasma noradrenaline level in music and excessive increase in noise stimulated group is observed compared to control. * indicates comparison of control with music and noise and # indicates comparison between music and noise. *** p≤0.001; ^###^p≤0.001.

The plasma corticosterone levels (nmol/l, mean ± SD) of PH1 chicks in control, music and noise stimulated groups were found to be 24.3±4.29, 24.62±3.7, 26.16±3.46 in males and 33.62±3.46, 35.40±4.09, 35.85±3.16 in females, respectively. Intra group comparison between males and females with Student’s t-test showed that females had significantly high corticosterone level as compared to males in control (t = −9.25, p≤0.001), music (t = −10.69, p≤0.001) and noise (t = −11.20, p≤0.001) stimulated groups (Fig. 4A). As there was significant difference between male and female plasma corticosterone level in all the groups, their values were not pooled and compared separately. Statistical analysis by one way ANOVA followed by post hoc Bonferroni test showed that there was no significant alteration (males: F (2, 87) = 2.003, p≤0.14; females: F (2, 87) = 2.70, p≤0.07) in the plasma corticosterone level in either sex, following chronic prenatal music or noise stimulation at 110 dB as compared to control (Fig. 4A).

### Plasma Noradrenaline Level

The plasma concentration of noradrenaline (ng/ml, mean ± SD) of PH1 chicks in control, music and noise stimulated groups were found to be 31.71±5.88, 52.64±7.79, 85.54±9.06 in males and 29.49±4.65, 47.83±5.27, 83.32±5.31 in females, respectively. It was evident from the data that the plasma noradrenaline level was increased following both types of sound stimulation but at the same time it is noteworthy that the increase was moderate in music (Mean difference with control: Male = 20.93, Female = 18.33) and excessive in noise (Mean difference with control: Male = 53.83, Female = 53.82) stimulated group as compared to the control (Fig. 4B). Statistical analysis by one way ANOVA showed that the increase was significant among the three groups (Male: F (2, 42) = 200.91, p≤0.001; Female: F (2, 42) = 467.06, p≤0.001). Further post hoc analysis with Bonferroni test confirmed that the music stimulated chicks showed significantly elevated (p≤0.001) plasma noradrenaline level as compared to the control whereas in the noise stimulated chicks, the level was even higher as compared to control (p≤0.001) and music (p≤0.001) stimulated chicks (Fig. 4B). Intra group comparison with Student’s t-test showed no significant alteration of plasma noradrenaline level between males and females in any group (Control: t = 1.1, p≤0.28; Music: t = 1.91, p≤0.07; Noise: t = 0.79, p≤0.44).

### Localization of Synaptophysin and PSD-95 by Immunohistochemistry

**Figure 5:**
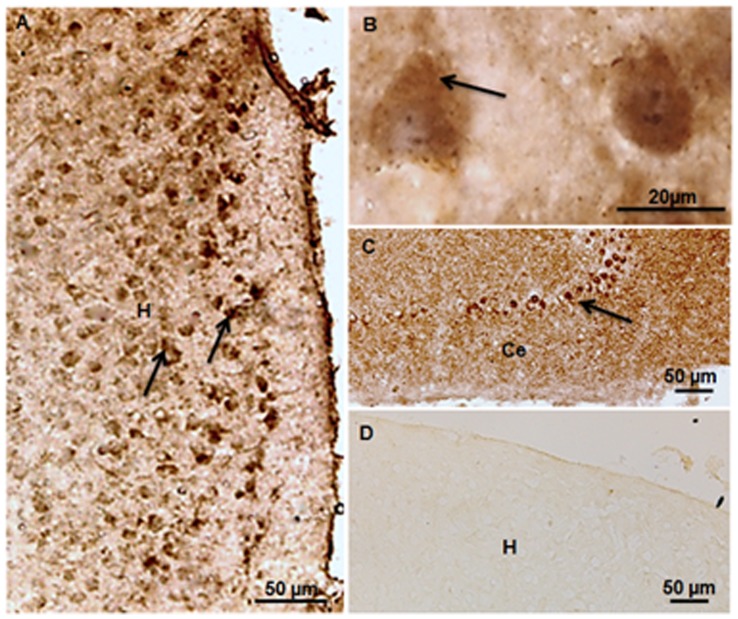
Immunolocalization of synaptophysin. (**A**) Photomicrograph of PH1 chick hippocampus shows synaptophysin immunoreactivity (arrows) in the control group. (**B**) Cytosolic expression of synaptophysin (arrows) is seen at higher magnification. (**C**) Cerebellum used as positive control, shows synaptophysin immunopositive Purkinje cells (arrows) at PH1. (**D**) Photomicrograph of hippocampus, showing lack of immunopositivity (negative control). H = Hippocampus; Ce = Cerebellum. Scale bars = 50 µm (**A, C, D**); 20 µm (**B**).

**Figure 6:**
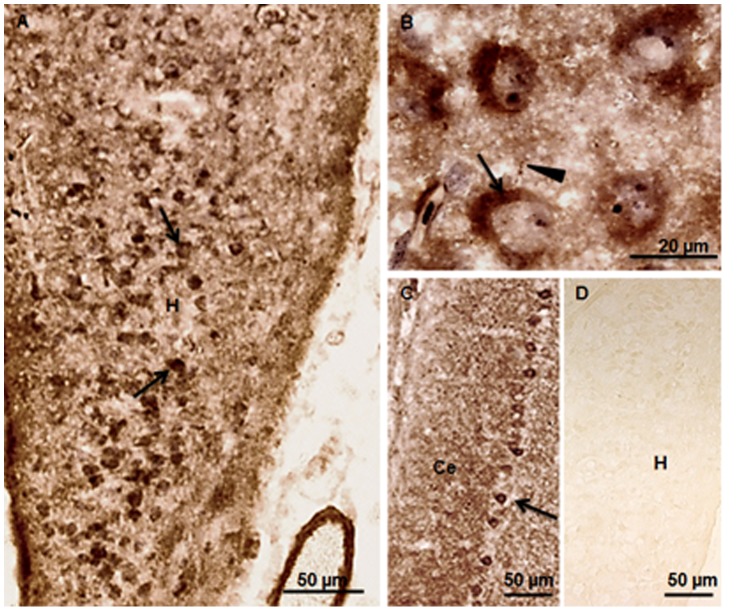
Immunolocalization of PSD-95. (**A**) Photomicrograph showing PSD-95 immunoreactivity (arrows) in the hippocampus of control chicks at PH1. (**B**) PSD-95 immunolocalization in the cytosol (arrows) and in the terminal boutons (arrowheads) in the neuropil of the hippocampus region. (**C**) Positive control shows immunostaining of the Purkinje cells (arrows) in the chick cerebellum. (**D**) Photomicrograph of hippocampus, showing lack of immunopositivity (negative control). H = Hippocampus; Ce = Cerebellum. Scale bars = 50 µm (**A, C, D**); 20 µm (**B**).

The synaptophysin (Fig. 5A) and PSD 95 (Fig. 6A) immunopositive neurons were evenly distributed throughout the dorso-ventral extent of chick hippocampus in control and the two auditory stimulated groups. The immunoreactivity was found in the cytosol of neurons (Fig. 5B, 6B) as well as in terminal boutons (Fig. 6B) in the neuropil of the hippocampus region. Chick cerebellum used as positive control showed immunopositive Purkinje cells stained with synaptophysin (Fig. 5C) and PSD-95 (Fig. 6C). The specificity of the immunoreactions and any alteration in the expression of both proteins were confirmed by Western blotting.

### Quantitation of Synaptophysin and PSD-95 by Western Blotting

**Figure 7:**
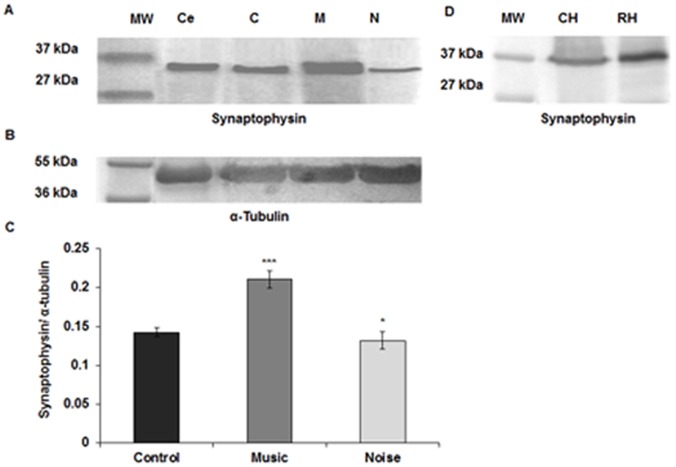
Western blot and densitometric analysis for synaptophysin. (**A**) Immunoblot shows levels of synaptophysin (37 kDa) in the chick hippocampus at PH1. The lane next to the molecular weight markers shows synaptophysin protein in chick cerebellum at PH1 as positive control. (**B**) α-tubulin used as loading control, is seen as bands of equal intensities at 55 kDa. (**C**) Histogram shows significant increase of synaptophysin in PH1 chick hippocampus of the music stimulated group and significant decrease in the noise stimulated group as compared to the control. (**D**) Both rat and chick hippocampus extracts show bands for synaptophysin at the same molecular weight level, confirming the specificity of the antibody to the same epitope *** p≤0.001; * p≤0.038. MW = Molecular weight marker, Ce = Cerebellum; C = Control; M = Music; N = Noise; CH = Chick Hippocampus; RH = Rat Hippocampus.

**Figure 8:**
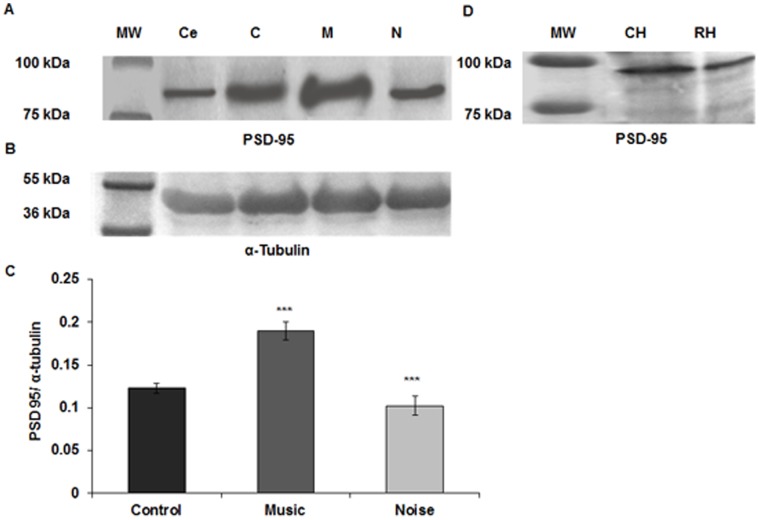
Western blot and densitometric analysis for PSD-95. (**A**) Immunoblot shows levels of PSD-95 (95 kDa) in the chick hippocampus at PH1. The lane next to the molecular weight markers shows PSD-95 protein in chick cerebellum at PH1 as positive control. (**B**) α-tubulin used as loading control, is seen as bands of equal intensities at 55 kDa. (**C**) Histogram shows significant increase of PSD-95 in PH1 chick hippocampus of the music stimulated group and significant decrease in the noise stimulated group compared to the control. (**D**) Both rat and chick hippocampus show bands for PSD-95 at the same molecular weight level, confirming the specificity of the antibody to the same epitope. *** p≤0.001. MW = Molecular weight marker, Ce = Cerebellum; C = Control; M = Music; N = Noise; CH = Chick Hippocampus; RH = Rat Hippocampus.

The Western blots for synaptophysin (Fig. 7A) and PSD 95 (Fig. 8A) showed bands at 37 and 95 kDa, respectively. It appeared that following both types of sound stimulation there were significant alterations in the expression of synaptophysin [F (2, 15) = 114.79, p≤0.001] and PSD-95 [F (2, 15) = 459.42, p≤0.001] among the three groups. Post hoc analysis with Bonferroni test further confirmed that the expressions of both the synaptic proteins were significantly higher (synaptophysin: p≤0.001, PSD-95: p≤0.001) in the music stimulated group while the noise group showed a considerable decrease (synaptophysin: p≤0.038, PSD-95: p≤0.001) as compared to control (Fig. 7D, 8D). α-tubulin was used as a loading control and showed bands of equal intensities in all the groups at 55 kDa (Fig. 7B, 8B). Immunoblots of both chick and rat hippocampal extracts showed corresponding bands at same molecular weight for each protein confirming the specificity of the antibodies to each protein (Fig. 7C, 8C).

### T maze Test

**Figure 9:**
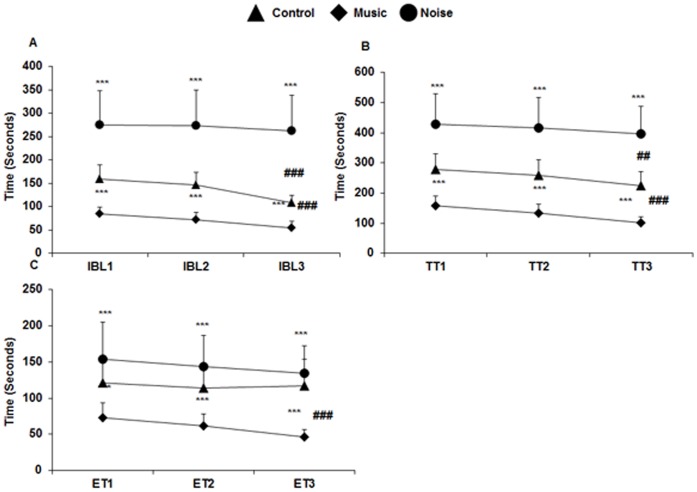
Effects of loud music and noise exposure on spatial orientation and learning. Line graphs, show the time (Mean ± SD) taken to exit the isolation box (**A**), total time (Mean ± SD) to reach the target (**B**) and exploration time (Mean ± SD) in the T-arm (**C**) in all the 3 trials in all the groups. Note the significantly less time taken by the chicks of music stimulated group and more time by the noise stimulated group in **A–C** in all the 3 trials as compared to the control. Inter-trial comparison shows significant reduction in time by the third trial in **A** and **B** in the control group and in **A–C** in the music stimulated group. Noise group does not show any alteration in time in successive trials (**A–C**). * indicates inter-group comparison and # indicates inter-trial comparison. *** p≤0.001; ## p≤0.014; ### p≤0.001. IBL = Isolation box latency; TT = Total time; ET = Exploration time; 1, 2, 3 denote trial number 1, 2 and 3.

i **Spatial orientation.** The ability of the PH1 chicks to navigate in a novel environment i.e. their spatial orientation was evaluated from the first trial of the T-maze test. Comparison of the first trial among the 3 groups by one way ANOVA showed a significant difference in the time taken to exit the isolation box i.e. IBL1 [F (2, 87) = 62.41, p≤0.001], exploration time i.e. ET1 [F (2, 87) = 19.59, p≤0.001] and the total time i.e. TT1 [F (2, 87) = 60.56, p≤0.001] to reach the target (Fig. 9). Further post hoc analysis confirmed that the music stimulated chicks had taken significantly less time to exit the isolation box as compared to control (p≤0.001) and noise (p≤0.001), whereas the noise stimulated chicks took significantly more time (p≤0.001) as compared to the control (p≤0.001) and music (p≤0.001) stimulated group (Fig. 9A). Similar trend was observed when the exploration time 1 (ET1) (Fig. 9C) and the total time 1 (TT1) (Fig. 9B) was compared among the three groups. Chicks from the music stimulated group traversed the maze faster as compared to the control (TT1: p≤0.001, ET1: p≤0.002) and noise (TT1: p≤0.001, ET1 p≤0.001), whereas the noise stimulated chicks were delayed in navigating the maze as compared to the control (TT1: p≤0.001, ET1: p≤0.04) and music (TT1: p≤0.001, ET1: p≤0.001) stimulated group (Fig. 9B–C).

It was evident from the above results that the spatial orientation was significantly improved in the music stimulated group, while the noise stimulation had a negative impact on the spatial orientation ability of PH1 chicks.

ii **Spatial learning.** The second and third trials of T maze were conducted to test the learning ability of the chicks. With each successive trial, the control group had taken significantly less time to exit the isolation box [F (2, 87) = 15.52, p≤0.001; Fig. 9A]; the total time to reach the target was also reduced [F (2, 87) = 4.53, p≤0.01; Fig. 9B]. Post hoc analysis showed that the decrease in the time spent in isolation box and the total time taken to reach the target was significant by the third trial (IBL: p≤0.001; TT: p≤0.014). However, the exploration time in the maze was not significantly reduced by successive trials in the control group [F (2, 87) = 0.19, p≤0.83; Fig. 9C).

In the music stimulated group, significant decline in the isolation box latency [F (2, 87) = 14.05, p≤0.001; Fig. 9A], total time [F (2, 87) = 14.23, p≤0.001; Fig. 9B] and exploration time [F (2, 87) = 9.91, p≤0.001; Fig. 9C] was observed in the subsequent trials. Further post hoc analysis confirmed that the decline in time in all the three parameters was significant by the third trial (IBL: p≤0.001; TT: p≤0.001; ET: p≤0.001; Fig. 9A–C).

Contrastingly, in the noise stimulated group no significant reduction in the time taken to exit the isolation box [F (2, 87) = 0.13, p≤0.88; Fig. 9A], the total time [F (2, 87) = 0.42, p≤0.66; Fig. 9B] and the exploration time [F (2, 87) = 0.42, p≤0.5; Fig. 9C] was observed in the successive trials.

**Figure 10:**
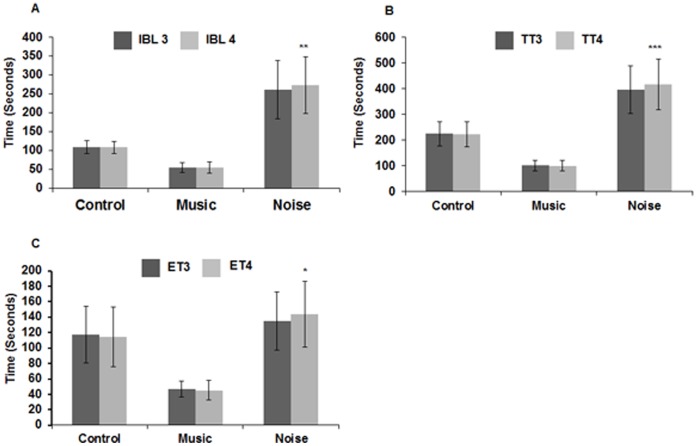
Effects of loud music and noise exposure on spatial memory. Histograms showing the time (Mean ± SD) taken to exit the isolation box (**A**), total time (Mean ± SD) to reach the target (**B**) and the exploration time (Mean ± SD) in the T-arm (**C**) during the third and fourth trial (24 h after the third trial). No significant alteration is observed between the trials in control and music stimulated group (**A–C**). Note that the time taken by the noise stimulated chicks is significantly increased in the fourth trial compared to the third for all the three parameters (**A–C**). *** p≤0.001; ** p≤0.004; * p≤0.03. IBL = Isolation box latency; TT = Total time; ET = Exploration time; 3, 4 denote third and fourth trial.

The above results clearly show that both control and music treated chicks performed better in the T-maze with each successive trial. On the other hand, in the noise stimulated group, learning was found to be impaired with no significant reduction in time in the successive trials.

iii **Spatial memory.** The ability of the chicks to memorize the learnt task was evaluated by repeating the T maze test (fourth trial) 24 h after the third trial (Fig. 10). Statistical analysis by paired t-test showed no significant difference between the third and the fourth trial in any of the three parameters, both in control (IBL: t = 0.32, p≤0.75; TT: t = 1.57, p≤0.14; ET: t = 1.2, p≤0.25) and music (IBL: t = −0.157, p≤0.88; TT: t = 0.503, p≤0.62; ET: t = 0.63, p≤0.54) stimulated group (Fig. 10A–C). In contrast, the noise stimulated chicks had taken significantly longer time in the fourth trial as compared to the third trial in all the three parameters (IBL: t = −3.46, p≤0.004; TT: t = −5.6, p≤0.001, ET: t = −2.37, p≤0.03; Fig. 10A–C). It was noted from the memory test that both the control and music stimulated chicks were able to memorize the task, whereas the noise stimulated chicks showed an impairment.

### Open Field Test

The data of all the groups was log transformed due to high variation and then analyzed. No significant difference amongst the groups was observed in the total distance moved, inner and outer zone stay duration and latency of first entry. However, the frequency of entry in outer zone was significantly different amongst the groups (F (2,85) = 3.36, p≤0.04), with noise group showing a trend for an increase. There was also a significant difference in the total time spent moving (F (2,86) = 4.30, p≤0.02) amongst the groups. Post hoc analysis of the total time spent moving revealed a significant increase (p≤0.03) in the noise group as compared to the music group. These results suggest an increase in locomotor and exploratory behavior in the noise group (Table 1).

**Table 1:** Data (mean±SD) of open field test conducted in control, music and noise exposed chicks.

	Groups			
Parameters	Control	Music	Noise	p value
Total distance Moved (cm)	625.81±539.55	587.66±637.68	855.91±850.37	0.62
Total time spent moving (s)	88.65±73.56	54.74±60.73*	126.8±121.39**#**	0.02
Inner Zone stay duration (s)	317.67±212.26	371.92±213.06	269.94±176.28	0.44
Inner Zone entry frequency	5.06±6.07	5.53±6.39	8±6.37	0.08
Outer Zone stay duration (s)	273.94±210.33	228.18±213.02	324.46±175.88	0.12
Outer Zone entry frequency	4.77±6.32	4.96±6.61	8±6.42	0.04
Outer Zone latency of first entry (s)	138.2±162.70	117.20±148.48	133.83±132.61	0.45

* indicates comparison of control with music/noise group and # indicates comparison of music with noise group. */# p<0.05.

## Discussion

The present study revealed that prenatal music stimulation at 110 dB sound pressure level significantly improved the spatial orientation as well as the spatial learning and memory of PH1 chicks while noise at the same sound pressure level negatively affected these abilities. Additionally, an enhanced expression of synaptic markers: synaptophysin and PSD-95 was observed in the hippocampus of PH1 chicks following music stimulation, while the noise resulted in a considerable decrease of these levels. We found significant alterations in plasma noradrenaline of both the auditory stimulated groups with a moderate increase in the music and an excessive increase in the noise stimulated group as compared to the control. On the other hand, no change was observed in the plasma corticosterone level in any of the experimental groups.

In humans as well as in birds, the environmental factors can influence the development of brain throughout the embryonic life [3]. Del Giudice [62] has reported that depending on the external illumination and mother’s abdominal thickness, the light that reaches the human fetus during the late gestation period can provide prenatal visual experience which could contribute to differential visuo-motor abilities in newborns. Studies by Rogers et al. [1], [3], [63] have confirmed the crucial role of prenatal visual stimulation in the development of chick thalamofugal visual pathway. In a similar way, auditory stimulation exerts a profound effect on the development of auditory areas as well as hippocampus of chicks (20, 52). Earlier studies from our laboratory have shown improvement in spatial orientation [18] and learning [18], [22] of neonatal chicks after exposure to prenatal species-specific as well as non species-specific sound (music) stimulation at 65 dB, suggesting an early maturation of neural connectivity. In the present study, the beneficial effects of music on learning and memory were seen even with higher sound pressure level of 110 dB. Interestingly, noise at the same sound pressure level delayed the spatial learning and impaired the spatial memory. The most convincing reason behind the cognitive enhancing effect of music or rhythmic sound is found to be physiological arousal [40], which is mediated probably by the release of hormones such as adrenocorticotropin, glucocorticoids, vasopressin and noradrenaline [38], [64]. In the present study, we found that the plasma noradrenaline level was moderately increased in music stimulated group as compared to the control, whereas in the noise group it was excessively increased. Concurrently, spatial behavior showed facilitation in the music stimulated group and retardation in the noise stimulated group. It is demonstrated that in rats, moderate increase of brain noradrenaline due to moderate foot-shock enhances memory retention, whereas excessive increase of noradrenaline due to high intensity of foot-shock results in memory retardation [65]–[67]. It is also known that noradrenaline level increases in different brain regions both after learning and stressful events [67], [68], but interestingly while its minimal increase facilitates cognitive performance, its high levels dampen the same [41]. Noradrenaline at higher concentration inhibits memory formation by the activation of α_1_ -adrenergic receptors, whereas a moderate level facilitates memory formation through β-adrenergic receptors [69]. In the present study, the moderate increase of noradrenaline in the music stimulated group may have acted via β-receptors to facilitate learning and memory in PH1 chicks. On the contrary, in the noise group, the retardation of the cognitive abilities may be a consequence of excessive noradrenaline level, working via α_1-_adrenergic receptors.

Noradrenaline is also known as a marker of stress and it is established that while severe stress negatively affects the performance of animals in different learning tasks [70]–[74], mild stress facilitates the same in young and adult animals [75]–[77]. The observed improvement in spatial learning and memory in music treated chicks could be due to the mild stress following rhythmic music stimulation, whereas the excessive stress in arrhythmic noise treated group could have resulted in impaired spatial learning and memory of PH1 chicks. Moreover, studies have shown that stress associated physiological changes also involve “behavioral inhibition” which is a fearful response towards a novel environment [78], [79] and noradrenaline plays an essential role in anxiety and behavioral inhibition [80]. High level of noradrenaline (increased arousal) can result in increased alertness in a novel environment which together with behavioral inhibition leads to a state of anxiety [80], [81]. The poor learning of PH1 chicks in the noise treated group in the present study may also result from an increase in baseline behavioral inhibition consequent to excessive increase in plasma noradrenaline level.

The spatial learning can also be affected by locomotor ability and exploratory potential of the animal. In rats, preweaning enrichment and in chicks, rearing for 3–5 days in an enriched environment facilitates their motor behavior [82] and spatial exploration [83]. Thus to discriminate between locomotor, exploratory drive and true spatial learning effects, the open field test was conducted. A significant increase in total time spent moving and frequency of entry into outer zone represents enhanced locomotion and exploration in the noise group as compared to the music group. In the T-maze test also, the noise stimulated chicks showed longer exploratory time but with a significant impairment of spatial learning suggesting that these movements were random and not goal directed. Thus facilitation of spatial learning in the music group may be due to better spatial orientation and target directed movements.

In both control and auditory stimulated groups, the female chicks showed significantly higher corticosterone level as compared to males. This sexual difference in plasma corticosterone level is evident in different species, including mammals, fishes, amphibians and birds [84], [85] and our results also confirm the same. However, as found in this study, the effect of prenatal chronic auditory stimulation either by music or noise on plasma corticosterone level was insignificant in both sexes. Previously it was reported that chronic exposure to unpredictable noise or restraint stress does not affect plasma corticosterone level in mouse and female rats, respectively [86], [87] and the most likely explanation for this is found to be the habituation effect [88]. Thus, in the present study, the insignificant alteration of corticosterone in PH1 chicks suggests a possible adaptation of the embryos to the sound that was given chronically for 11 days.

The presynaptic protein synaptophysin is involved in vesicular exocytosis [89], [90] and is a reliable marker of synaptogenesis [91]–[94]. Enriched environment is known to increase synaptophysin level in the hippocampus and other cortical regions in different animals including neonatal chicks [18], [47], [95]. An enhanced expression of synaptophysin is suggested to enhance the cognitive abilities in different animals [96]–[98]. Contrastingly, in Alzheimer’s patients, impaired cognitive function is correlated with reduced synaptophysin immunoreactivity in the hippocampus and frontal cortex [99]–[101]. Impairment in learning and memory is also observed in synaptophysin knockout mice [102], indicating an important role of synaptophysin in modulating these cognitive abilities. In the present study, we found an enhanced expression of synaptophysin in the hippocampus of music stimulated chicks, whereas the expression was greatly reduced in noise treated hippocampus. The increase in synaptophysin level in the music stimulated group may suggest increased synaptogenesis in the hippocampus which can contribute to the improved learning and memory in PH1 chicks. On the contrary, the observed decrease in expression of synaptophysin in the hippocampus following noise stimulation could be due to the reduced synaptogenesis and responsible for the impaired learning and memory of PH1 chicks.

The role of PSD-95 in the maturation and stabilization of activity-dependent excitatory synapses is well established [51], [103]. The enhanced expression of PSD-95 is known to facilitate long term potentiation [104] and remodeling of dendritic spines [51], [105]–[107], suggesting a possible role of PSD-95 in regulating neuronal morphology and synaptic plasticity [108]. PSD-95 mutant mice showed variations in dendritic spine density in the striatum and hippocampus [109] and also impaired spatial learning in water maze [110]. In the present study, PSD-95 showed enhanced expression in the music stimulated group whereas the expression was significantly compromised in the noise treated group as compared to the control. This differential expression of PSD-95 in the hippocampus thus may play a crucial role in neuronal plasticity and hence can be a reason behind the observed altered learning efficacy and memory of PH1 chicks. Thus, the difference in the expressions of these synaptic proteins in the music and noise treated group may provide a biochemical basis for the observed altered learning and memory of PH1 chicks.

The importance of rhythmic nature of music or any sound in facilitating learning and memory is well documented in the postnatal life [36], [111], [112]. Studies have shown that while exposure to a complex rhythmic stimulus has the potential to enhance long-term memory consolidation in one day old chicks, the background noise, isochronous stimulus, white noise and human environmental noise do not facilitate retention [36]. Exposure to non-rhythmic music in mice produced impairments on a spatial learning task with associated abnormal branching of hippocampal neuronal dendrites and excessive glial growth, whereas no such effects were observed following exposure to synchronized music [113]. Field et al. [64] have demonstrated that memory could be enhanced by ethologically relevant rhythmic maternal hen calls but not with non-rhythmic maternal hen calls, indicating that ethological relevance is not a sufficient condition for this effect. These studies suggest that rhythmicity is the fundamental element of any sound that augments the cognitive behavior in animals. In our study, the sound pressure level of both the auditory stimuli (music and noise) presented, was same at 110 dB but other physical properties such as wave pattern and sound energy distribution were disparate. The complex wave form of the music stimulus with variations in wave length/tones was rhythmic in nature due to the repetition of a particular pattern with time. In contrast, the noise stimulus with its simple wave form and a constant wave length was continuous and arrhythmic. Furthermore, in the present study, despite the overlapping frequency range and same sound pressure level, the energy distribution of the music and noise stimuli was different. In the music stimulus the high sound energy was distributed throughout the frequency range whereas in the noise it was centered at 2.7 kHz. These differences in the physical properties of the music and noise stimuli may contribute to the observed biochemical changes and the differential behavioral outcome in the two experimental groups. Further studies are needed to throw light on the contribution of the individual characteristics of music and noise in producing the observed outcome, as reported in the present study.

Till date several studies regarding the effect of rhythmic sound stimulation on cognitive abilities have been performed where music stimulation was given at a moderate sound pressure level (60–65 dB). This is the first study to evaluate the effects of prenatal music stimulation at a significantly higher decibel (110 dB), which is usually considered harmful for cognition. Interestingly, current findings suggest that despite the high decibel, music stimulation, when provided prenatally, can significantly improve the learning and memory of neonatal chicks. The present study also reports for the first time the possible detrimental consequences of prenatal noise exposure on the spatial behavior of newly hatched chicks.

It is accepted that use of animal model in the present study restricts its direct application on humans, but the sounds (music and noise) used in the present study are the ones that we are surrounded with in our daily life. It is noteworthy that both chicks and humans are precocious i.e., they can hear extraneous sounds during embryonic/fetal stage and the ability to memorize the auditory cues is well developed in birds as it is in humans [34]. In natural environment chicks are attracted to those auditory stimuli that are repetitive, segmented and have short component notes [114] and chicks perceive the pattern of complex rhythmic stimulus that are similar to chicken maternal calls due to comparable rate and similarities in frequency range [36], [114]. It has been earlier reported that chicks exposed to prenatal species-specific calls, slow music, or fast music show preference to maternal calls in the auditory preference test [17] which indicates their preference to the ethologically relevant sounds. In humans too the newborns are able to distinguish their mother’s voice and prefer to respond to the maternal sound [115], [116]. An extensive review by Kaplan [34] described that bird’s rhythmic performance to music is quite similar to human musical rhythmic behavior and their ability to distinguish different styles and rhythms is not elementary rather well refined and sophisticated. The ability to perceive musical genre is observed in pigeons [117] and Java sparrows [118] which suggests that there is great similarity in the way the birds and humans hear music [119]. Hence any prenatal sound exposure may influence the human fetuses in a similar way as it affects the neonatal chicks. Thus, the current study provides an insight into the possible consequences of *in-utero* exposure to high decibel rhythmic and arrhythmic sounds on the spatial and cognitive abilities of human newborns.
